# Identification of Novel A2/C2 Inter-Genotype Recombinants of Hepatitis B Virus from a Korean Chronic Patient Co-Infected with Both Genotype A2 and C2

**DOI:** 10.3390/ijms18040737

**Published:** 2017-03-30

**Authors:** So-Young Lee, Seung-Hee Lee, Ji-Eun Kim, Hong Kim, Kijeong Kim, Yoon-Hoh Kook, Bum-Joon Kim

**Affiliations:** 1 Department of Biomedical Sciences, Microbiology and Immunology, Liver Research Institute and Cancer Research Institute, College of Medicine, Seoul National University, 28 Yongon-dong, Chongno-gu, Seoul 110-799, Korea; leesy8822@snu.ac.kr (S.-Y.L.); edgeleading@naver.com (S.-H.L.); nell3566@snu.ac.kr (J.-E.K.); wild0804@snu.ac.kr (H.K.); yhkook@snu.ac.kr (Y.-H.K.); 2 Department of Microbiology, School of Medicine, Chung-Ang University, Seoul 156-756, Korea; kimkj@cau.ac.kr

**Keywords:** Hepatitis B virus, polymerase RT region, small surface protein, subgenotype A2, subgenotype C2, inter-genotypical recombination

## Abstract

Nearly all cases of Hepatitis B virus (HBV) infections in South Korea have the C2 genotype. Here, we have identified a chronically infected patient who was co-infected with HBV of both the A2 and C2 genotypes by screening 135 Korean chronically infected patients using direct sequencing protocols targeting the 1032-bp polymerase reverse transcriptase (RT) region. Further polymerase chain reaction (PCR)-cloning analysis (22 clones) of the RT showed that this patient had genotype C2 (12 clones), genotype A2 (six clones) and A2/C2 inter-genotype HBV recombinants (four clones). BootScan analysis showed that three of the four recombinants have different types of recombination breakpoints in both the RT and overlapping hepatitis B surface antigen (HBsAg) region. Given the significance of HBsAg as a diagnostic or vaccination target against HBV infection, clinical implications of these identified recombinants should be studied in the future. To our knowledge, this is the first report on A2/C2 inter-genotype HBV recombinants.

## 1. Introduction

Hepatitis B virus (HBV) is considered a high-risk factor for chronic liver disease, which can lead to cirrhosis and hepatocellular carcinoma [1,2,3,4]. It has been reported that in addition to more than 350 million people being chronically infected with HBV, the annual number of deaths caused by HBV-related diseases [5], including cirrhosis and hepatocellular carcinoma (HCC), is estimated to be approximately 786,000 worldwide [6]. According to the Korean National Health and Nutrition Survey of 2013, the prevalence of the HBV surface antigen (HBsAg) was 3.9% [7], which classifies Korea as an endemic area for HBV.

HBV is an enveloped DNA virus and a member of the Hepadnaviridae family. The HBV genome virion consists of incomplete double-stranded circular DNA, which is approximately 3.2 kb long. The genome contains four overlapping open reading frames (ORF) that encode the surface antigen (S), X protein (X), core (C) and polymerase (P), which has reverse transcriptase activity. The envelope forms part of the surface of the virus particle, which is recognized as HBsAg. HBsAg is one of the long open reading frames. However, it has triple start codons that encode preS1, preS2, and S. The polypeptides with three different sizes are called large (preS1, preS2 and S), middle (preS2 and S) and small (S). The reverse transcriptase (RT) of the HBV polymerase consists of 344 amino acids and partially overlaps with the small surface protein (HBsAg). For this reason, mutations in the RT region could result in alterations in the replication capacity, antigenicity, encapsulation, tolerance against antiviral therapy and virulence of HBV [8,9,10]. 

According to the criteria of 8% divergence in the complete genome sequence, HBV strains have been classified into eight genotypes designated as A–H [11,12,13]. The HBV genotype can affect the clinical features of infected patients and responses to antiviral therapies [14,15]. Furthermore, the isolated frequency of each genotype from patients chronically infected with HBV differs depending on the geographic area of the world [16,17]. For these reasons, determining the genotype of HBV is critical for establishing a therapeutic strategy in chronically infected patients. Notably, previous studies have reported that genotype C2, which is more virulent and has a lower antiviral response than genotype B [18,19], has an extraordinarily high prevalence in South Korea [20,21]. In addition, there is a high incidence of the basal core promoter (BCP) double mutation in Korea, which has been rarely seen in other countries. The high incidence of this mutation could cause the development of unique HBV variants arising from chronically infected patients [22]. In particular, various types of unique HBV variants are known to be associated with the progression of liver disease, including HCC, in South Korea [20,21,23,24,25,26,27,28,29,30,31,32,33,34,35,36,37,38,39,40,41].

Inter-genotype recombination is regarded as an important element of HBV genetic variability and may impose challenges on vaccine designation and antiviral therapy strategies [42,43]. The high prevalence of vertical infections in HBV endemic areas, such as South Korea, can lead to a life-long chronic infection [44], which can result in a high probability of co-infection and a high risk for virus recombination. However, other genotypic or inter-genotypic infections have rarely been reported to date in South Korea, possibly due to the exclusive distribution of genotype C2 [21]. In this study, we report on the first novel HBV recombinants in the polymerase RT region between genotypes A2 and C2 in a chronically infected patient from South Korea.

## 2. Results

### 2.1. Identification of Inter-Genotypic Recombinants of Genotype A2/C2 from a Korean Patient, H62, by Phylogenetic Analysis of 1032-bp Polymerase RT Sequences

**Figure 1:**
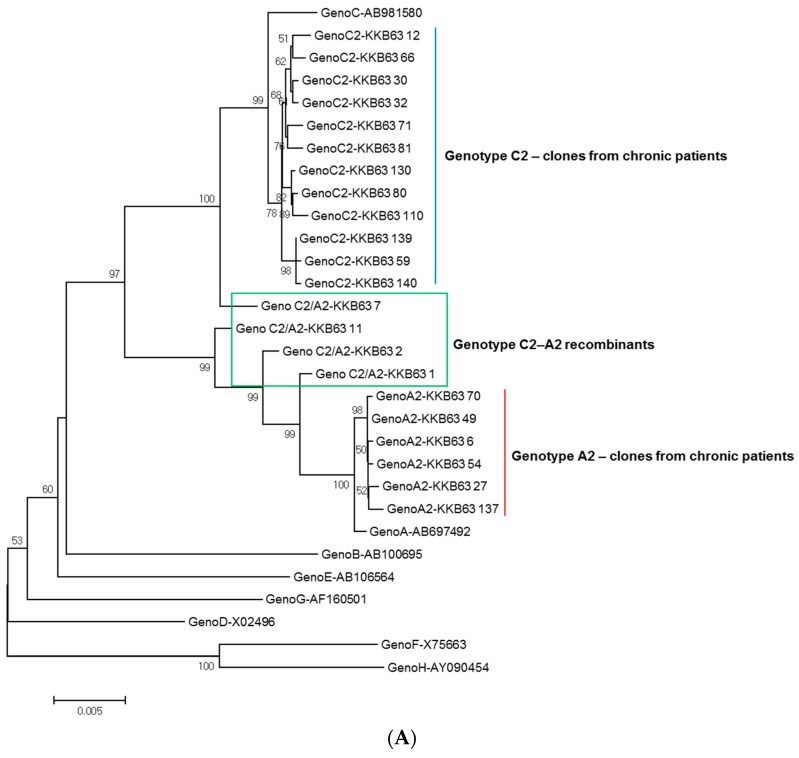

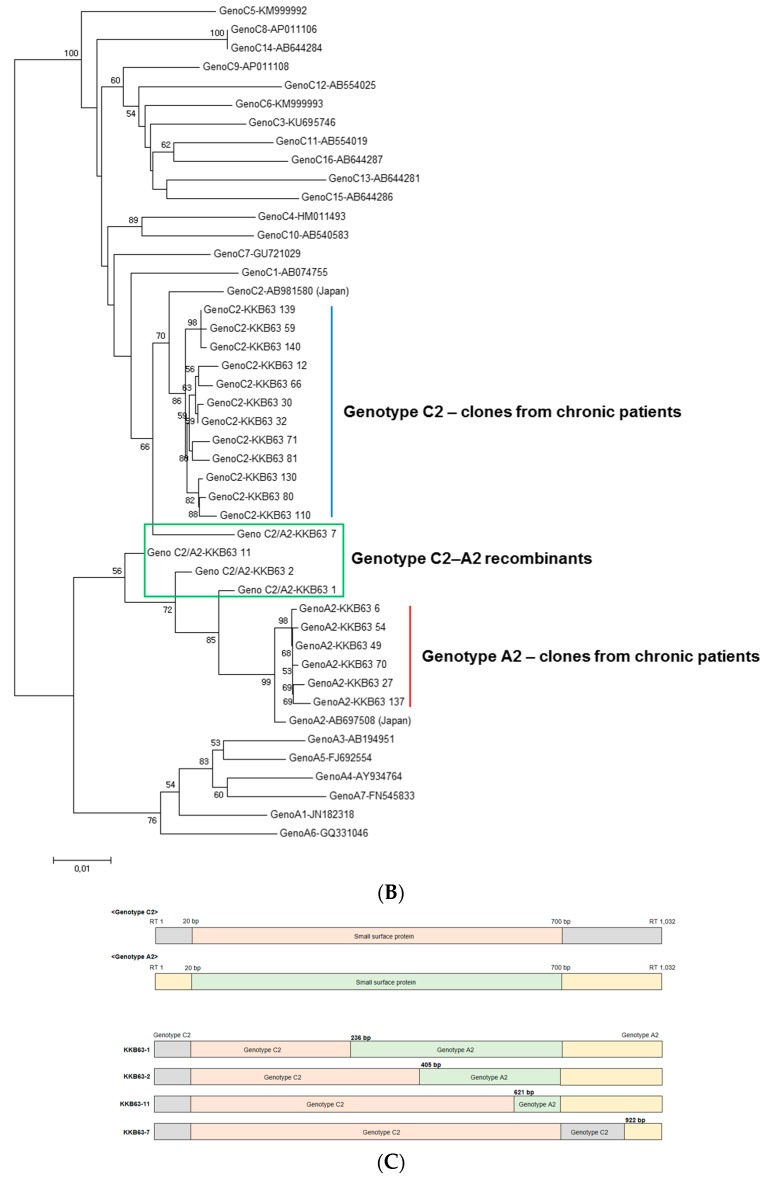
Phylogenetic analysis of 22 clones from a Korean chronically infected patient based on the 1032-bp reverse transcriptase (RT) region and schematic representation of the RT and the overlapping hepatitis B surface antigen (HBsAg) regions showing recombination profiles of the four genotype A2/C2 recombinants. Phylogenetic relationships of 22 clones were compared with (**A**) genotype A–H reference strains and (**B**) sub-genotypes of genotype A and C based on the RT gene (1032 bp). The bootstrap values were calculated from 1000 replications and Bootstrap values of <50% are not shown. (**C**) Analysis of alignments revealed that the RT region overlaps the HBsAg region (21–700 bp). The four recombinants contained different types of recombination breakpoints. The recombination break points of KBB63-1, KBB63-2, KBB63-11 and KBB63-7 are located at the positions nt 236, nt 405, nt 621 and nt 922, respectively.

The phylogenetic analysis based on 1032-bp sequences covering the entire RT region for genotyping showed that 131 samples (97.0%) out of 135 belonged to genotype C2, with three samples and one sample belonging to genotypes A and B, respectively (data not shown). One patient, KKB63, showed mixed sequence profiles in the electropherogram of direct sequencing (data not shown). To verify the exact sub-genotyping of this patient, we further analyzed the quasi-species distribution of 22 polymerase chain reaction (PCR) clones of the RT region from patient KKB63 using a PCR-cloning protocol. Their phylogenetic determination into sub-genotypes was performed using sequences of reference strains of genotype A or C sub-genotypes (Figure 1A). It is important to note that further phylogenetic analysis of 22 clones of the RT region showed that this patient had genotype C2 (12 clones), genotype A2 (six clones) and A2/C2 inter-genotype HBV recombinants (four clones) (Figure 1B). The strains belonging to genotype A2 (6 clones) and C2 (12 clones) were separated into their respective sub-genotypes, which showed a high level of sequence similarity with the reference strains. Six clones of genotype A2 and 12 clones of genotype C2 had a similarity value of more than 97.5% and 97.2% compared to the respective reference strains, respectively. Phylogenetic separation into sub-genotypes was also checked using deeper analysis of the sub-genotype–specific signature sequences within the polymerase RT region [45,46]. Six signature sequences for genotype A2 (E356, W501, T607, I617, H619 and K666) and nine signature amino acids for genotype C2 (V449, N467, T474, N485, K495, F497, K500, R620 and K/Q679) were conserved in the clones of the patients (Figure S1).

However, our phylogenetic study indicated that the four recombinant clones were clustered into different branches, which were distinct from each other and those of genotypes A2 and C2. Similarity values of these recombinant clones ranged from 92.7% to 98.5% and from 92.6% to 97.3%, when compared to the reference strains of genotypes A2 and C2, respectively. It suggests that the four recombinants have distinct recombination profiles.

### 2.2. Identification of Recombinant Breakpoints in Four Recombinant Clones by BootScan Analysis

To analyze the recombinant profiles of these four recombinants, BootScan analysis was performed. It revealed that all four recombinants had similar recombination structures with a C2 genotype backbone at the amino terminal of the RT and a recombined A2 genotype region at the carboxyl terminal of the RT (Figure 1C). The possible recombination break points in KKB63-1, KKB63-2, KKB63-11 and KKB63-7 were located at positions nt236, nt405, nt621, and nt922, respectively. In three of the recombinant clones (KKB63-1, KKB63-2 and KKB63-11), the recombination breakpoints were included in the overlapping HBsAg region, meaning that these recombinations could affect the structure and function of the HBsAg and the RT region. However, this was not the case in the other recombinant clone, KKB-7.

**Figure 2:**
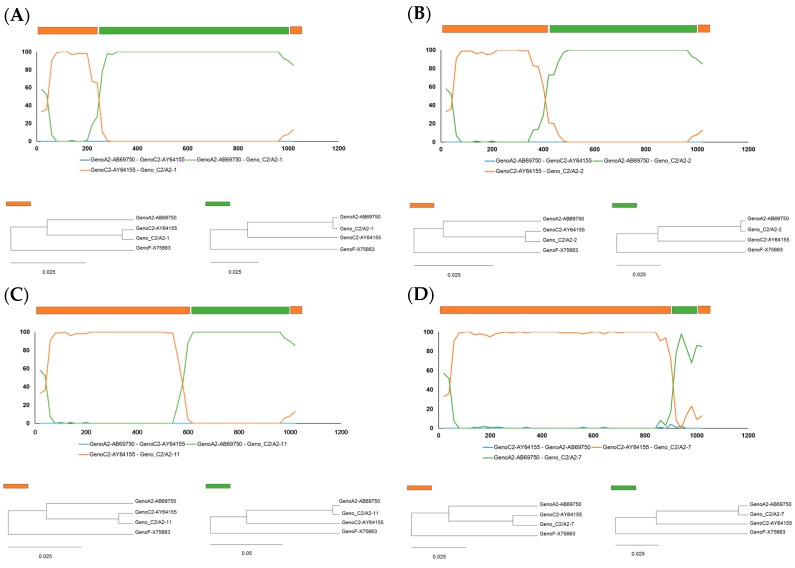
Recombination detection program (RDP) analysis and phylogenetic relationship of four recombinants, (**A**) KKB63-1; (**B**) KKB63-2; (**C**) KKB63-11; and (**D**) KKB63-7. The possible recombination fragment of KKB63-1 was showed nt 264–1017, with KKB63-2, KKB63-11 and KKB63-7 having possible recombination segments as nt 405–1017, nt 621–1017 and nt 922–1018, respectively. All of the recombination fragments were clustered into genotype A2, although the possible parental fragments were clustered into genotype C2. These results were analyzed by RDP4 with the automated BootScan method, with high significance found for five different algorithms. The phylogenetic tree of the two fragments from each recombinant clone were grouped with genotype F.

To further define the precise sub-genotypes of the recombinant fragment, all the recombinant clones were analyzed for the phylogenetic distances of both possible parental and recombination fragments (Figure 2). Our data showed that all four recombinants had possible parental fragments of the RT amino terminal and recombination fragments of the RT carboxyl terminal that were clearly clustered into genotype C2 and genotype A2, respectively, further supporting the above finding that the RT sequences of all the recombinants are from recombination events of genotypes C2 and A2. The recombination was evaluated by nine different algorithms with recombination detection program (RDP). There were highly significant values for recombination in five of these nine methods, which were the GENECONV, BootScan, MaxiChi, Chimaera and PhylPro methods (Figure 2).

### 2.3. The Simultaneous Modification of HBsAg by Recombination in the Polymerase RT Region

**Figure 3:**
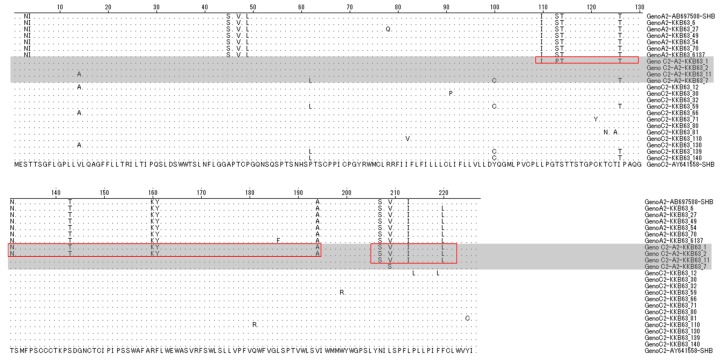
Alignment of the overlapping HBsAg regions of the 22 clones and the reference strains of genotypes A2 and C2. The three recombinants KKB63-1, KKB63-2 and KKB63-11 had recombination breakpoints in HBsAg at the amino acid positions 110, 131 and 207, respectively. The amino acids which matched with the reference strain of genotype C2 were hidden. The alignment was performed by MegAlign software with Jotun Hein Method (Windows. Version 3.12e, DNASTAR, Madison, WI, USA).

To check whether the RT recombination could lead to simultaneous changes in HBsAg, its putative 227 amino acids in the four recombinant clones were compared with the other clones and the reference strains of genotypes A2 and C2. As expected, three recombinant clones (KKB63-1, KKB-2 and KKB-1) were shown to have recombination breakpoints in the overlapping HBsAg region and to have three distinct types of chimeric HBsAg sequences, depending on the location of the recombination breakpoints. The N terminals and C terminals of these sequences are from genotype C2 and genotype A2, respectively. KKB63-1, with a breakpoint at position nt 236, resulted in a chimeric HBsAg for which the N-terminal region from amino acids 1–109 and the C-terminal region from amino acids 110–227 are from genotypes C2 and A2, respectively. KKB63-2, with a breakpoint at position nt 405, led to a chimeric HBsAg for which the N-terminal region from amino acids 1–130 and the C-terminal region from amino acids 131–227 are from genotypes C2 and A2, respectively. KKB63-11, with a breakpoint at position nt621, led to a chimeric HBsAg for which the N-terminal region from amino acids 1–206 and the C-terminal region from amino acids 207–227 are from genotypes C2 and A2, respectively. However, KKB63-7, with no breakpoints in the overlapping HBsAg region, led to HBsAg with only a C2 genotype, not a chimeric one (Figure 3). 

## 3. Discussion

HBV infection is a global health issue leading to chronic liver disease and it has geographical differences. HBV has been classified into eight genotypes, based on differences in nucleotide sequences. The intergroup nucleotide divergence of approximately between 4% and 8% of the full-length genome within genotypes has led to further division into sub-genotypes. HBV genotypes A–D, F and I have been classified into various sub-genotypes [47]. However, in the HBV genotypes E, G and H, no sub-genotypes have been introduced to date. The nine and 16 sub-genotypes described so far are from the HBV genotype A and genotype C, respectively. 

Vertical infection is a major transmission route in endemic areas, such as South Korea, causing life-long chronic infection and leading to the presence of different genotypes in the same area. As a result of this vertical infection, there is a high probability of co-infection in a host [44]. In addition, co-infection dramatically increases the risk of virus recombination [48,49]. Previous studies have reported various inter-genotype recombinations between genotypes A and D [50], A and E [45], A and G [51], B and D [42,52], in addition to C and D [53] in different geographical regions having a co-circulation of different genotypes. However, inter-genotypic recombination between genotype A and C has rarely been reported to date. In particular, recombination between the A2 and C2 genotypes found in this study has not been described previously to the best of our knowledge. To date, only four HBV sequences showing the A/C inter-genotypic recombination have been introduced so far, namely AY057947 from China (Tibet), AY233277 from South Africa and EF494376 and EF494378 from Taiwan [53,54,55]. We further checked recombination profiles between sub-genotypes of genotypes A and C. Among these, AY057947 and EF494376 had a genotype C1 genome integrated with a genotype A2 fragment in the pre-S1/S2 and pre-C/C, respectively. EF494378 showed a genotype C5 genome integrated with a genotype A2 fragment in the S–X region. AY233277 was a recombination of genotype A1 and a small genotype C15 fragment in the X and pre-C parts. Among these, only EF494378 from Taiwan proved to have the recombination breakpoint in the overlapping HBsAg region, as shown in our three recombinant clones, KKB63-1, KKB-2 and KKB-11 (data not shown).

The amino acid residues coding for the d/y and w/r serotypes are located at positions 122 and 160 of the HBsAg [11]. To date, a total of 10 different serotypes of HBsAg (ayw1, ayw2, ayw3, ayw4, ayr, adw2, adw3, adw4q−, adrq−, and adrq+) have been described [18]. Among our four recombinants, three strains with the recombination breakpoint in the overlapping HBsAg region (KKB63-1, KKB-2 and KKB-11) could produce chimeric HBsAg sequences, subsequently affecting their serotype determination. For this reason, we analyzed serotypes of 22 clones by comparing the amino acid sequences of their HBsAgs. Our 12 genotype C2 clones and six genotype A2 clones proved to be adrq– and adw2 serotypes, respectively. It is important to note that two recombinants, KKB63-7 and KKB63-11, proved to be adrq− subtypes, as shown in other genotype C2 clones. The other two recombinants, KKB63-1 and KKB63-2, proved to be adw2 subtypes, as shown in other genotype A2 clones (data not shown). This suggests that recombination in the HBsAg region could affect the HBV serotype, which could have clinical significance in addition to potentially affecting the vaccine efficiency. 

HBV genotype A is widespread in northern Europe, Africa and India [17], but is not commonly found in Asia, which has a high prevalence of genotype C [56,57]. However, according to recent reports, the prevalence of HBV genotype A has increased in Japan, with this unusual distribution potentially being caused by globalization and sexual transmission [58]. Although nearly 100% of all infections in South Korea have been genotype C2 [20,21], our phylogenetic analysis found that three patients out of 135 (2.2%) were infected with genotype A2 (data not shown), reflecting the recent increase in genotype A2 infections in South Korea. For this reason, it is tempting to speculate that the recent increase in globalization or the influx of North Korean defectors could affect the HBV genotype distribution in South Korea, which subsequently could lead to the generation of possible novel recombinants of HBV.

In conclusion, we have discovered novel A2/C2 HBV recombinants from a chronically infected Korean patient in this study. Particularly, three of four recombinants have recombination breakpoints in the overlapping HBsAg region, which produced chimeric versions of HBsAg. Given the significance of HBsAg as a diagnostic or vaccination target against HBV infection, the clinical implications of these identified recombinants should be studied in the future. To our knowledge, this is the first report on A2/C2 inter-genotype HBV recombinants.

## 4. Materials and Methods

### 4.1. Patient

In this study, a chronically infected patient, KKB63, was verified to be infected with recombinant HBV from a cohort of 135 serum samples. These samples were collected from patients diagnosed to be chronically infected with hepatitis, who were followed at the Digestive Disease Center of Konkuk University Hospital, Korea between March 2008 and January 2011. This study was approved by the Institutional Review Board of Konkuk University Hospital (KUH-1010544) and Seoul National University Hospital (IRB-1605-065-761). This patient was a 45-year-old woman diagnosed with chronic hepatitis B virus. The HBV DNA and alanine aminotransferase levels of this patient were 3.29 (log_10_ copies/mL) and 22 IU/L, respectively. The patient tested positive for the hepatitis B surface antigen (HBsAg) but negative for hepatitis B envelope antigen (HBeAg).

### 4.2. DNA Extraction and Amplification of the Polymerase RT Region

DNA was extracted from the serum of patients with a QIAamp DNA Blood Mini kit (QIAGEN, Hilden, Germany) and dissolved in 20 μL of Tris-EDTA (TE) buffer (10 mM Tris-HCl, 1 mM ethylenediaminetetraacetic acid (EDTA), pH 8.0). First-round PCR was performed with the sense primer POL-RT-F1 (5′-CAG CCT ACT CCC ATC TCT CCA CCT CTA AG-3′) and antisense primer POL-RT-R1 (5′-GCT CCA GAC CGG CTG CGA GC-3′) to yield a 1375-bp amplicon between positions nt 3157 and nt 1316 of the HBV genome. Second-round PCR was performed with the sense primer POL-RT-F2 (5′-CCT CAG GCC ATG CAG TGG AA-3′) and antisense primer POL-RT-R2 (5′-GTA TGG ATC GGC AGA GGA GC-3′) to yield a 1291-bp amplicon between positions nt 3196 and nt 1271 of the HBV genome. The PCR mixture contained 1.5 mM MgCl_2_, 200 μL of deoxynucleotide triphosphates (dNTPs) and 2.6 U of Expand High Fidelity Taq polymerase. For the PCR amplification, templates were denatured at 95 °C for 10 min, before 30 cycles of 95 °C (45 s), 52 °C (45 s) and 72 °C (90 s) were completed. The final extension was done at 72 °C for 5 min. The PCR products of 135 patients were subject to direct sequencing analysis

### 4.3. TA Cloning and Sequencing Analysis of the Amplicon

The PCR products from a chronically infected patient, KKB63, were purified with the MEGA quick-spin Fragment DNA purification kit (Intron biotechnology, Seoul, Korea) and cloned into the TOPO TA cloning vector (Invitrogen, Carlsbad, CA, USA). The nucleotide sequences were sequenced with the dideoxy method using the BigDye Terminator Cycle Sequencing Ready reaction V.2 and a fluorescent 373A DNA sequencer (Applied Biosystems, Foster City, CA, USA). 

### 4.4. HBV Genotyping 

For genotyping of 135 HBV strains, their 1032-bp RT sequences determined by direct sequencing were compared with the sequences of eight reference strains representing each of the genotypes (A–H including the C2 strains) obtained from GenBank. For further genotyping of 22 clones from a chronically infected patient, KKB63, their RT sequences were also compared with nineteen reference strains of genotype A2 and C2. The sequences of the polymerase RT region were compared by phylogenetic/molecular evolutionary analysis with MEGA version 4.0 [59]. Phylogenetic trees were obtained with the neighbor-joining method (1000 bootstrap replicates), with the mean genetic distances being estimated by Kimura two-parameter analysis [60,61]. 

### 4.5. BootScan Analysis

The sequences of the four A/C inter-genotype recombinant clones were compared with the reference strains of genotype A2 and C2 using the RDP version 4.66 [62]. The identification of possible recombination events and parental sequences were done with RDP, GENECONV, BootScan, MaxChi, Chimaera, SiScan, 3Seq, Likelihood Analysis of Recombination in DNA (LARD) and PhylPro methods implemented in the RDP4 program with automated BootScan analysis. The graphics of the position in the alignment were drawn, based on the plot data in the Microsoft Excel Program. In addition, the RDP4 program also provided phylogenetic trees of the sequences for major parental regions as well as for possible recombination events.

## 5. Conclusions

In conclusion, we reported novel recombinants of HBV genotype C2/A2 from a chronically infected patient in South Korea co-infected with both genotypes A2 and C2 in this study. Notably, three of the four RT recombinants also produced three types of distinct recombinant chimeric HBsAgs of genotype A2/C2. Given the significance of HBsAg as a diagnostic or vaccination target against HBV infection, further investigations should be done to clarify the epidemiological traits, clinical implication and virological characteristics of the novel A2/C2 inter-genotype recombinant HBV.

## Supplementary Materials

Supplementary materials can be found at www.mdpi.com/1422-0067/18/4/737/s1.
