# Supplementary material for: Identification of Novel A2/C2 Inter-Genotype Recombinants of Hepatitis B Virus from a Korean Chronic Patient Co-Infected with Both Genotype A2 and C2
Source: Int J Mol Sci. 2017 Mar 30;18(4):737. doi: 10.3390/ijms18040737 (PMC5412322; doi:10.3390/ijms18040737)
Supplement: Supplementary file 1 [file ijms-18-00737-s001.pdf]

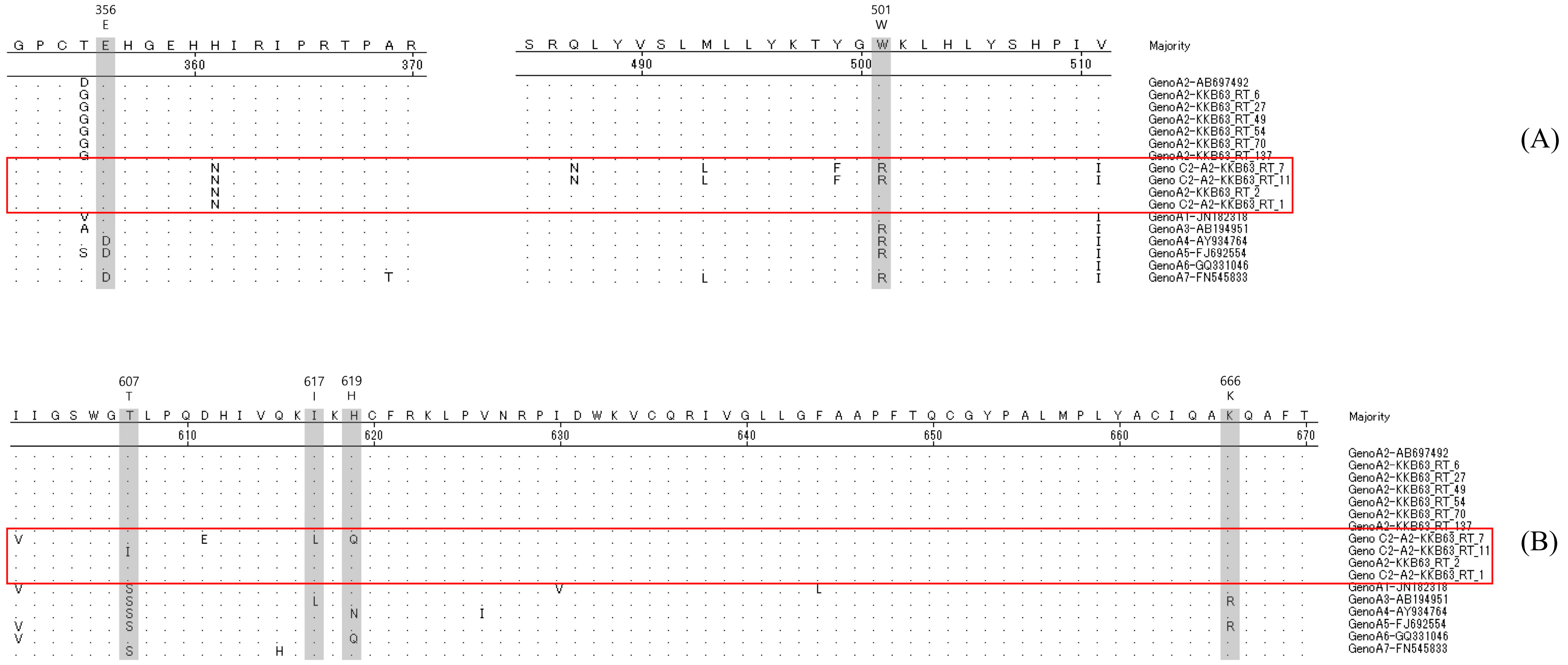

Figure S1. Alignment of reference strains and clones of sub-genotypes of (**A**) genotype A and (**B**) genotype C based on RT region. (**A**) The sub-genotype specific amino acids of genotype A were conserved in six-clone and sub-genotype A2. (**B**) The same specific signatures were shown in 12 clones and reference strains of sub-genotype C2. The specific signatures of the amino acids were recorded, with the 4 recombinants marked with red squares. The amino acids which matched with the reference strain of genotype A2 or genotype C2 were hidden. The alignment was performed by MegAlign software with the Jotun Hein Method
